# Adenocarcinoma in situ detected on a thin-walled lung cavity: a case report

**DOI:** 10.1186/s40792-022-01413-w

**Published:** 2022-04-04

**Authors:** Takashi Sakai, Yoko Azuma, Satoshi Koezuka, Hajime Otsuka, Atsushi Sano, Naobumi Tochigi, Akira Iyoda

**Affiliations:** 1 Division of Chest Surgery, Department of Surgery, Toho University School of Medicine, 6-11-1, Omorinishi, Ota, Tokyo, 143-8541 Japan; 2 Department of Surgical Pathology, Toho University School of Medicine, Tokyo, Japan

**Keywords:** Cavitary lung carcinoma, Lung cancer, Adenocarcinoma in situ

## Abstract

**Background:**

Cavitary lesions pathologically diagnosed as adenocarcinoma in situ (AIS) have been rarely reported. The examination of these type of lesions is necessary for a better understanding of the mechanisms underlying their formation and development of more efficient diagnostic and treatment strategies. Here, we present the case of a patient with cavitary lung carcinoma, diagnosed as AIS, who underwent partial resection.

**Case presentation:**

A 72-year-old man presented with an abnormal shadow on chest radiography. Computed tomography findings showed a nodule in the right upper lobe, which was later diagnosed as an adenocarcinoma via transbronchial biopsy. A thin-walled cavity with partial thickening in the right lower lobe was also noted. We suspected that the thin-walled cavitary lesion was malignant, and performed wedge resection during a right upper lobectomy. AIS was diagnosed based on the histopathological findings of the thickened part of the thin-walled cavity.

**Conclusions:**

This study highlights that, although rare, AIS may be observed in cavitary lung carcinoma cases, particularly in thin-walled lesions.

## Background

Cavitary lung carcinoma is a common cancer with an incidence rate of 16%. However, carcinomas of the thin-walled lung cavity, particularly those measuring ≤ 4 mm, are rare [1, 2]. Cavitary lung carcinoma was reported to be more malignant and have poorer prognosis than non-cavitary lung carcinoma, and cavitary lesions pathologically diagnosed as adenocarcinoma in situ (AIS) have been rarely reported [1, 3–6]. We report the case of a patient with cavitary lung carcinoma, which was diagnosed as AIS, who underwent partial resection.

## Case presentation

**Fig. 1:**
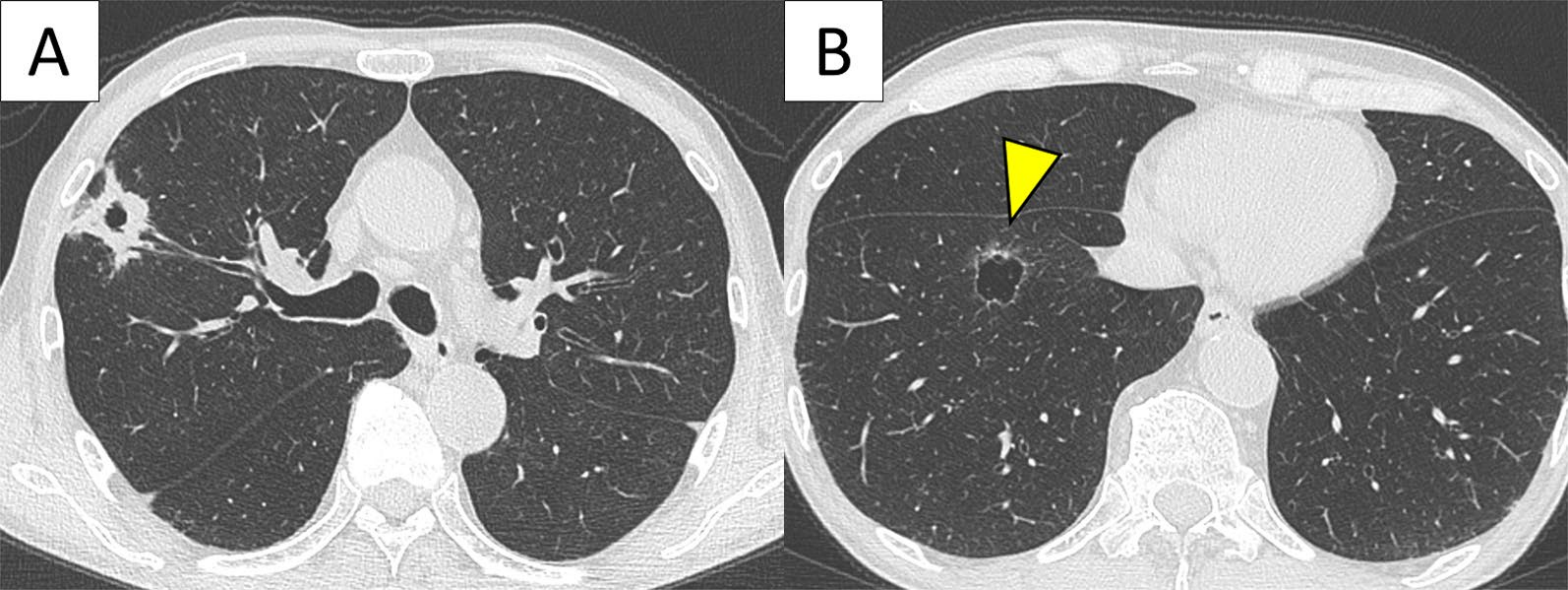
Imaging findings. **a** Computed tomography (CT) findings showing a 2.9 × 2.0-cm nodule on the right-upper lung. **b** CT scan also shows a thin-walled cavitary lesion with partly thickened areas on the right-lower lobe (arrow indicated the partly thickened cavitary wall)

A 72-year-old man was referred to our hospital after an abnormal shadow was observed in chest radiographic findings. He had been smoking 1.5 packs per day for 35 years, had been treated for chronic obstructive pulmonary disease, and had recently undergone a surgery for duodenal carcinoma, pT3N0M0 Stage IIA 3 months prior. Computed tomography (CT) findings revealed a 2.9 × 2.0-cm solid nodule with a cavity on the right upper lung (Fig. 1a). Positron emission tomography (PET)–CT scan revealed marked fluorine-18 deoxyglucose (^18^F-FDG) accumulation with a maximum standardized uptake value (SUV_max_) of 7.94. Levels of all tumor markers related to the lung carcinoma were within the normal range. This nodule was diagnosed as a primary adenocarcinoma via transbronchial biopsy. Another 2.2 × 1.7-cm thin-walled cystic lesion was also observed in the right lower lung (Fig. 1b), with weak ^18^F-FDG accumulation with an SUV_max_ of 1.68. The thickness of the lesion’s wall was approximately 1 mm, but it was partially thickened to 2 mm. Furthermore, an ill-defined ground glass area was defined around the thickened part. Thus, we presumed that the cystic lesion was secondary lung carcinoma, and resected the lesion during the right upper lobectomy. We performed a wedge resection to preserve lung function and prevent postoperative complication, and the surgical margin was sufficient with a length of at least 10 mm.

**Fig. 2:**
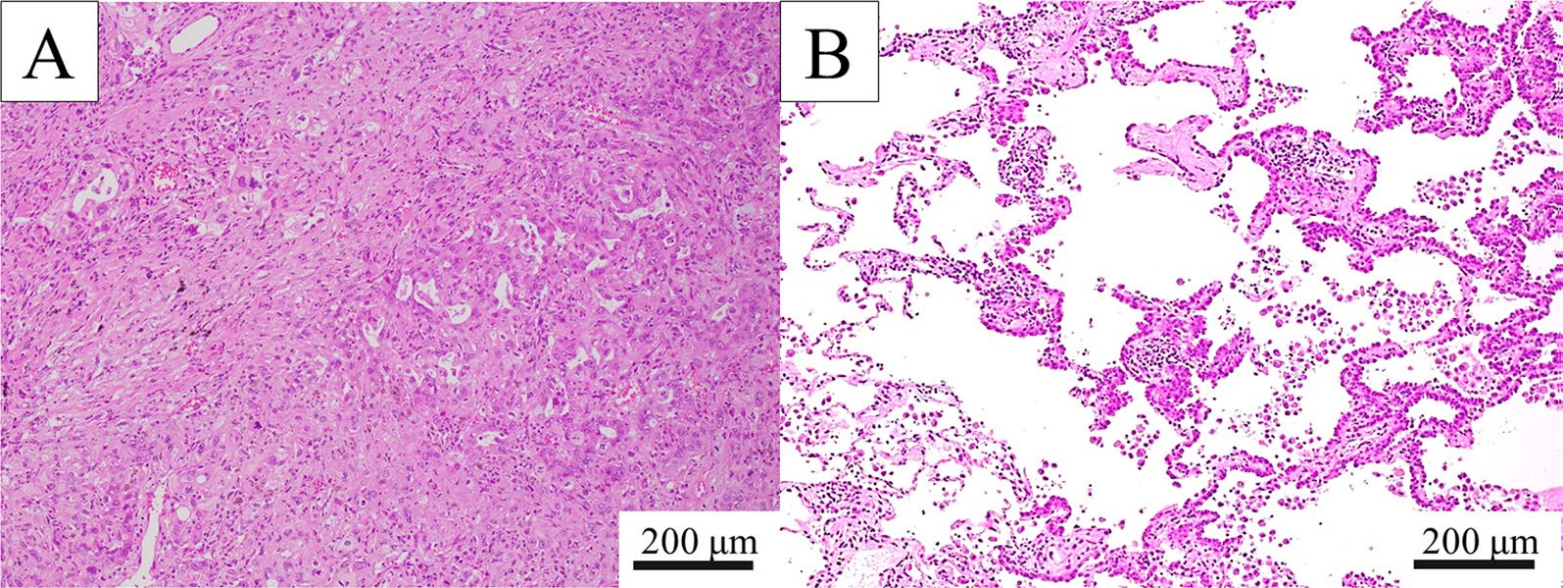
Pathological findings. **a** Tumor cells proliferated with solid, papillary, and lepidic growth patterns in the right-upper lung adenocarcinoma (hematoxylin and eosin method, ×100). **b** Adenocarcinoma in situ detected in the right-upper lung cavitary lesion (hematoxylin and eosin method, ×100)

**Fig. 3:**
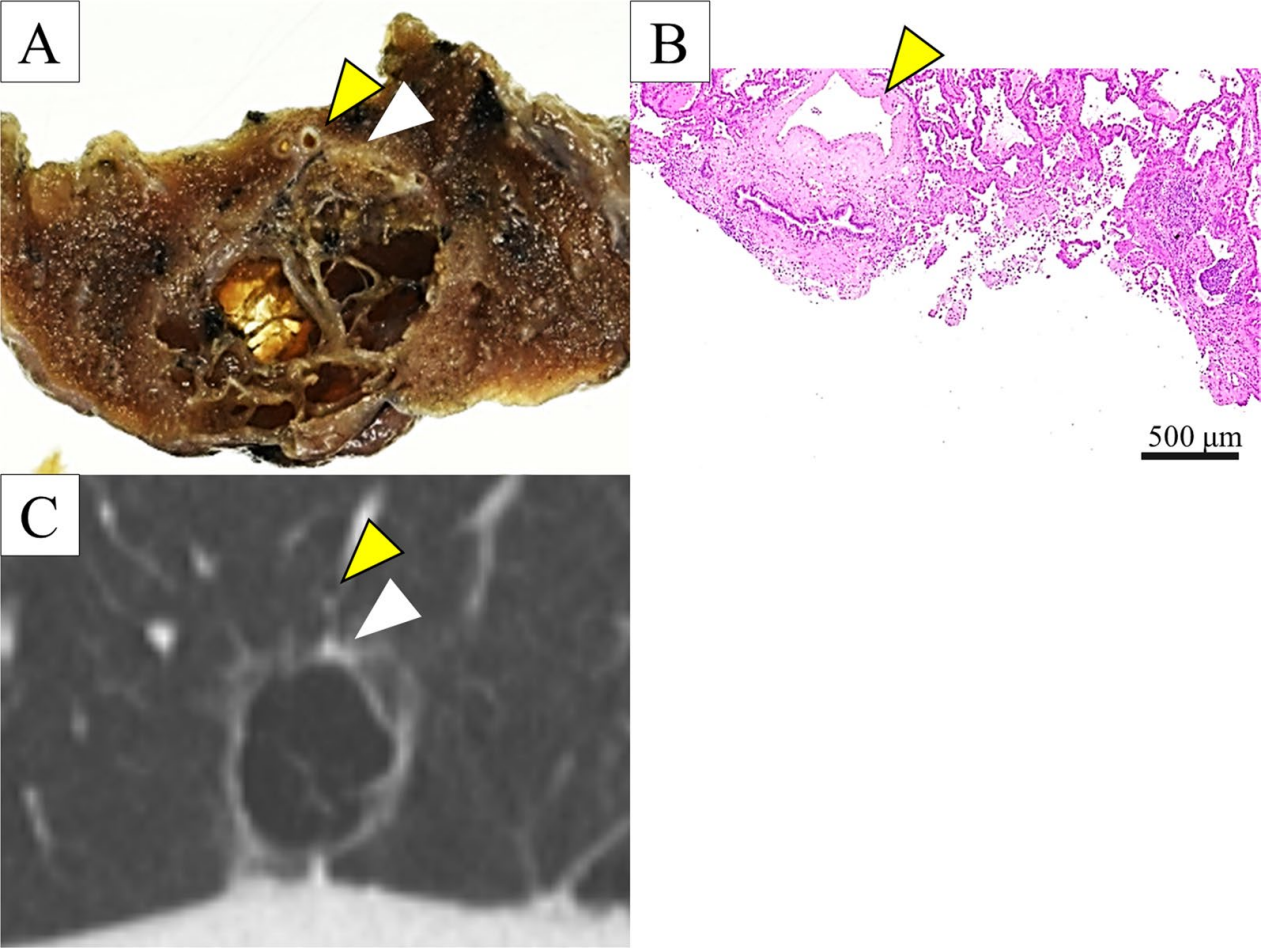
Detailed analysis of the histopathological and computed tomography scan findings suggestive of check-valve mechanism of cavity formation. **a**, **b** Yellow arrows show the bronchi flowing into the cavitary lesion. **a** White arrow indicates the adenocarcinoma in situ (AIS) detected at the partly thickened area of the cavity. **b** Microscopic findings showing that the AIS is surrounded the bronchi. **c** AIS detected on the histopathological findings is consistent with the partly thickened wall of the cystic lesion

Following histopathological examination, the right upper lung nodule was diagnosed as an adenocarcinoma with solid, papillary, and lepidic subtypes (Fig. 2a). It was classified as a pT3N1M0 Stage IIIA tumor due to the intrapulmonary and lymph node metastasis detected in the right upper lobe. Furthermore, the AIS proliferated on the part of the right-lower cavitary lesion (Fig. 2b). Therefore, the patient was diagnosed with multiple synchronous lung adenocarcinomas. Detailed analysis of the imaging and histopathological findings of the thin-walled cavitary lesion revealed that the AIS surrounded the bronchi flowing into the lesion (Fig. 3a, b), which was consistent with the thickness detected on the CT scan (Fig. 3c). These findings suggested that the mechanism of cavity formation might have been a check-valve system secondary to the AIS obstructing the bronchus.

## Discussion

There are various mechanisms of cavitary lung cancer formation, with tumor necrosis or abscess due to ischemia involving the feeding vessels and obstruction of bronchioles being the most common one [1]. Other etiologies include carcinogenesis of the cavitary wall, destruction of the alveolar wall by the protease or mucin produced by the tumor, and the check-valve mechanism due to the infiltration of the cancer into the bronchiole [1, 5, 7]. The aforementioned mechanisms of cavity formation are associated with the high malignancy potential of cavitary lung cancer [4, 5].

Histological examination of the surgical specimen from our patient did not reveal inflammatory cell infiltration and vasculitis in the cavity or lungs. This suggested the absence of necrosis and abscess in the tumor. In addition, other than the AIS, no other histopathological findings related to cancer-developing origin were observed in the cavity wall. CT findings showed a thickening of the cavity wall located at the end of the bronchus flowing into the cystic lesion, and histological examination revealed that the AIS surrounded the bronchus. It was unclear whether the AIS obstructed the airflow, and it is possible that it coincidentally occurred on the cyst wall. The etiology of the present cavity formation may be attributed to a check-valve mechanism, as described in previous reports [5, 8].

The frequency of detection may increase with the improvement of diagnostic imaging techniques. If cavitary wall thickness is detected, cancerous changes such as an ill definition, ground glass, and vascular convergence should be evaluated using thin-sliced or high-resolution CT scan. Moreover, clinicians should consider performing a PET–CT to screen for malignancy. If malignancy is suspected, even in thin-walled lesions, patients should be closely monitored for lesion growth. Furthermore, surgical biopsy should be considered to attain a definitive diagnosis.

## Conclusions

Thin-walled cavitary lung cancer cases are reportedly rare. However, AIS may be detected, such as in the present case. Cancerous findings should be checked for when lung cavity is detected.

## Data Availability

Data sharing is not applicable to this article as no data sets were generated or analyzed during the current study.

## Abbreviations

AIS: Adenocarcinoma in situ
CT: Computed tomography
PET: Positron emission tomography
^18^F-FDG: Fluorine-18 deoxyglucose
SUV_max_: Maximum standardized uptake value
